# Seasonal Dynamics and Forecasting of Toscana Virus Infections in Italy, 2016–2027: A Modelling Study

**DOI:** 10.3390/epidemiologia7050120

**Published:** 2026-08-25

**Authors:** Omar Enzo Santangelo, Anna Sole Pizzamiglio, Carlotta Vella

**Affiliations:** Territorial Health Care and Social Agency of Lodi, Azienda Socio Sanitaria Territoriale di Lodi, 26900 Lodi, Italy; annasole.pizzamiglio@asst-lodi.it (A.S.P.); carlotta.vella@asst-lodi.it (C.V.)

**Keywords:** Toscana virus, infectious disease forecasting, predictive analytics, time series analysis, arbovirus, Italy, epidemiological modelling

## Abstract

**Background**: Toscana virus is a sandfly-borne phlebovirus and a relevant cause of central nervous system infections in Mediterranean countries, including Italy. Its incidence exhibits strong seasonal patterns, suggesting suitability for time series modelling approaches. This study aimed to compare statistical and time series models for describing and forecasting Toscana virus incidence in Italy. **Methods**: Monthly confirmed autochthonous Toscana virus cases in Italy (2016–2025) were obtained from national surveillance data. The series was split into a training period (2016–2023) and a validation period (2024–2025). Three models were evaluated: SARIMA(1,0,1)(1,0,0)_12_, Poisson regression with harmonic seasonal terms, and Negative Binomial regression accounting for overdispersion. Predictive performance was assessed using MAE and RMSE. The best model was re-estimated on the full dataset to generate forecasts for 2026–2027. **Results**: All models captured strong seasonality and temporal dependence. The SARIMA model showed the best predictive performance (MAE = 4.75; RMSE = 7.63), outperforming Poisson (MAE = 4.99; RMSE = 11.59) and Negative Binomial models (MAE = 5.39; RMSE = 13.57). The Negative Binomial model confirmed significant overdispersion (α = 0.143, *p* < 0.001) but did not improve forecasting accuracy. Forecasts indicated persistence of seasonal oscillations through 2027. **Conclusions**: Toscana virus incidence in Italy follows stable seasonal dynamics. SARIMA provided the most accurate forecasts and is the preferred model for short-term prediction.

## 1. Introduction

Toscana virus (TOSV) is a sandfly-borne phlebovirus belonging to the family *Phenuiviridae* and is recognized as one of the most prevalent arthropod-borne viruses causing human central nervous system infections in endemic regions of the Mediterranean basin [1]. Since its initial identification in Italy, TOSV has been increasingly recognized as an important etiological agent of aseptic meningitis and meningoencephalitis during the summer months, coinciding with peak activity of Phlebotomus sandflies [2].

Toscana virus (TOSV) is transmitted to humans primarily through the bite of infected phlebotomine sandflies, particularly species of the genus *Phlebotomus*, and its transmission is closely linked to the seasonal activity of these vectors [3]. Human infections are frequently asymptomatic or mild, but neuroinvasive disease can occur and may manifest as meningitis, encephalitis, or meningoencephalitis, making TOSV an important cause of central nervous system infections in Mediterranean countries [2,4]. In Italy, TOSV transmission is geographically heterogeneous and has been documented particularly in central and northern regions, with serological evidence indicating substantial population exposure in several endemic areas [5]. Occupational and environmental exposure may influence the risk of infection, particularly among adults and individuals living or working in rural or peri-urban settings where contact with sandflies is more likely [5]. Recent national surveillance data further indicate that neuroinvasive TOSV infections are predominantly reported among adults, particularly males and working-age individuals, and residence in rural areas is associated with increased infection risk [6]. Neuroinvasive TOSV infections have been subject to national notification in Italy since 2016, providing an important surveillance framework for characterizing temporal and seasonal patterns at the national level [6]. The combination of substantial clinical impact, seasonal transmission, and identifiable population-level risk patterns supports the development of forecasting approaches that may contribute to improved surveillance and seasonal preparedness.

Epidemiological studies have demonstrated that Toscana virus infections exhibit a marked seasonal pattern, with most cases occurring between late spring and early autumn, reflecting the ecological dynamics of the vector population [3]. In endemic areas such as Italy, Spain, and southern France, seroprevalence studies suggest widespread exposure in the human population, although a substantial proportion of infections remain asymptomatic or underdiagnosed [4].

Despite its clinical relevance, Toscana virus surveillance remains heterogeneous across countries and long-term temporal patterns are still insufficiently characterized. Previous research has highlighted the importance of climatic and environmental factors in shaping sandfly abundance and virus transmission dynamics, suggesting that TOSV incidence is strongly driven by seasonal and interannual variability [7]. These characteristics make Toscana virus a suitable candidate for time series modelling approaches aimed at capturing both short-term dependence and seasonal cycles.

In infectious disease epidemiology, statistical forecasting models play a crucial role in understanding transmission dynamics and supporting public health preparedness. Several statistical approaches have been applied to infectious disease count data, including Poisson and Negative Binomial regression models. Poisson regression is commonly used for modelling count outcomes, whereas Negative Binomial regression provides greater flexibility when the variance exceeds the mean, a common feature of infectious disease surveillance data. These regression-based approaches can also incorporate temporal, environmental, and demographic covariates, allowing the assessment of factors associated with disease incidence [8]. However, when strong temporal dependence and recurrent seasonal patterns are present, models that explicitly account for autocorrelation and seasonality, such as Seasonal Autoregressive Integrated Moving Average (SARIMA) models, may provide advantages for short-term forecasting. Generalized Additive Models (GAMs) represent another flexible approach for infectious disease modelling, particularly when nonlinear associations between disease incidence and temporal or environmental predictors are expected. By using smooth functions, GAMs can accommodate nonlinear trends and seasonal patterns without imposing a strictly linear functional form. They have therefore been increasingly applied to vector-borne diseases and other infections in which climatic and ecological drivers may act nonlinearly. In the present study, however, the primary objective was to compare forecasting approaches based on the temporal structure of the observed surveillance series. We therefore focused on SARIMA and count regression models, while recognizing that GAMs and other covariate-driven approaches represent important directions for future research, particularly if environmental and climatic predictors are incorporated. However, when temporal dependence and strong seasonality are present, stochastic time series models such as Seasonal Autoregressive Integrated Moving Average (SARIMA) models may provide superior predictive performance by directly modelling autocorrelation structures [9].

Although both classes of models have been applied in infectious disease forecasting, comparative evaluations in the context of Toscana virus remain limited. In particular, few studies have directly assessed the predictive performance of SARIMA models against count-based regression approaches for arboviral infections with pronounced seasonal dynamics.

Therefore, the aim of this study is to (i) characterise the temporal dynamics of Toscana virus infections in Italy from 2016 to 2025, (ii) compare the predictive performance of SARIMA, Poisson, and Negative Binomial regression models, and (iii) generate short-term forecasts for the period 2026–2027 using the best-performing model.

## 2. Materials and Methods

### 2.1. Data Source and Study Design

Monthly counts of notified autochthonous Toscana virus infections in Italy were extracted from official surveillance bulletins published by the Istituto Superiore di Sanità (National Institute of Health) on 28 June 2026 [10]. The study period was selected to cover the longest continuous period for which nationally reported Toscana virus surveillance data were available in a consistent format. The series begins in January 2016 because national surveillance of neuroinvasive Toscana virus infections in Italy was established in 2016, providing a well-defined starting point for systematic national case ascertainment [6]. The study period was extended through December 2025 because this represented the most recent complete calendar year available for analysis at the time of dataset construction. This 10-year period provided 120 consecutive monthly observations, allowing the characterization of recurrent seasonal patterns while retaining a recent epidemiological context relevant to short-term forecasting. The surveillance dataset comprised laboratory-confirmed autochthonous Toscana virus infections reported through the Italian national arbovirus surveillance system. In accordance with the surveillance data structure, cases were classified according to their epidemiological origin, and only autochthonous confirmed infections were included in the present analysis [10]. The monthly series contained several observations with zero reported cases, particularly during the winter and early spring months. These zero values were retained as valid observations and were not considered missing data. No missing monthly observations were identified over the study period, and therefore no imputation procedure was required. For model development and validation, the dataset was partitioned into a training sample (January 2016–December 2023) and an independent test sample (January 2024–December 2025). This temporal split allowed an out-of-sample assessment of predictive performance under realistic forecasting conditions. Three competing forecasting approaches were considered: a Seasonal Autoregressive Integrated Moving Average (SARIMA) model, a Poisson regression model, and a Negative Binomial regression model. All models were estimated using the training period and subsequently evaluated on the test period using forecast accuracy metrics. The best-performing specification was then re-estimated on the full dataset (2016–2025) and used to generate forecasts for January 2026–December 2027.

### 2.2. Seasonal Autoregressive Integrated Moving Average (SARIMA)

The SARIMA model captures both short-term temporal dependence and seasonal structure in the data. The general specification is given by:

$$\Phi_{P}(B^{s})\;\phi_{p}(B)\;(1-B{)}^{d}(1-B^{s}{)}^{D}y_{t}=\Theta_{Q}(B^{s})\;\theta_{q}(B)\;\varepsilon_{t},$$

where:

- B is the backshift operator;
- s=12 denotes monthly seasonality;
- p,d,q are the non-seasonal autoregressive, differencing, and moving average orders;
- P,D,Q are the seasonal autoregressive, differencing, and moving average orders;
- $\varepsilon_{t}∼N(0,\sigma^{2})$ is a white noise process.

Based on preliminary identification procedures, a SARIMA(1,0,1)(1,0,0)_12_ specification was selected:

$$(1-\phi_{1}B)(1-\Phi_{1}B^{12})y_{t}=(1+\theta_{1}B)\varepsilon_{t}.$$

All model parameters were estimated via maximum likelihood.

#### SARIMA Model Identification and Selection

Four candidate SARIMA specifications were evaluated: SARIMA(1,0,0)(1,0,0)_12_, SARIMA(1,0,1)(1,0,0)_12_, SARIMA(1,0,0)(1,0,1)_12_, and SARIMA(1,0,1)(1,0,1)_12_. Model identification was guided by inspection of the ACF and PACF, which indicated substantial short-term autocorrelation and a pronounced seasonal component at lag 12. Candidate models were estimated by maximum likelihood and compared using AIC and BIC. Although the more parsimonious SARIMA(1,0,0)(1,0,0)_12_ model showed the lowest information criteria, the candidate specifications were subsequently assessed using out-of-sample forecasting performance on the independent 2024–2025 validation period. The SARIMA(1,0,1)(1,0,0)_12_ specification was retained as the final forecasting model because it provided the lowest MAE and RMSE in the validation period (see Table 1). Residual ACF/PACF and Ljung–Box tests were additionally used to assess the adequacy of the selected model. No automated model-selection procedure was used.

### 2.3. Poisson Regression Model

Given that monthly notifications are count data, a generalized linear model with a log-link function was employed.

The outcome variable was assumed to follow a Poisson distribution:

$$Y_{t}∼Poisson(\lambda_{t}),$$

with conditional mean specified as:

$$log(\lambda_{t})=\beta_{0}+\beta_{1}{t+\beta }_{1}sin\left(\frac{2\pi t}{12}\right)+\beta_{2}cos\left(\frac{2\pi t}{12}\right)+\beta_{4}Y_{t-1}$$

The harmonic sine and cosine terms capture annual seasonal fluctuations, while the lagged dependent variable accounts for short-term temporal dependence.

### 2.4. Negative Binomial Regression Model

To account for potential overdispersion in the count process, a Negative Binomial model was also considered:

$$Y_{t}∼NB(\mu_{t},\alpha ),$$

with conditional mean:

$$log(\mu_{t})=\beta_{0}+\beta_{1}{t+\beta }_{1}sin\left(\frac{2\pi t}{12}\right)+\beta_{2}cos\left(\frac{2\pi t}{12}\right)+\beta_{4}Y_{t-1}$$

The corresponding conditional variance is given by:

$$Var(Y_{t})=\mu_{t}+\alpha \mu_{t}^{2},$$

where α is the overdispersion parameter estimated from the data.

### 2.5. Model Evaluation

Forecasts produced for the period January 2024–December 2025 were compared with observed values from the test sample. Given the relatively limited length of the monthly time series (120 observations), a fixed temporal hold-out design was retained as the primary validation strategy. The final 24 months of the series (January 2024–December 2025) were reserved as a completely out-of-sample validation period, corresponding to 20% of the available observations and covering two complete annual seasonal cycles. This approach allowed all competing models to be evaluated over the same independent forecasting horizon while preserving a sufficiently long training period for estimation of the seasonal and autoregressive components. A rolling-origin forecasting approach could provide additional information on the stability of predictive performance across different forecasting origins. However, given the limited length of the available series and the primary objective of evaluating short-term forecasting performance over a fixed independent horizon, rolling-origin validation was not implemented in the present study. This methodological consideration is acknowledged as a potential direction for future validation studies.

Predictive accuracy was assessed using two standard metrics:

Mean Absolute Error (MAE):

$$MAE=\frac{1}{n}\sum_{t=1}^{n}|y_{t}-{\hat{y}}_{t}|,$$

and Root Mean Squared Error (RMSE):

$$RMSE=\sqrt{\frac{1}{n}\sum_{t=1}^{n}(y_{t}-{\hat{y}}_{t}{)}^{2}}.$$

The model achieving the lowest MAE and RMSE was selected as the optimal forecasting specification.

### 2.6. Final Forecasting Model

The SARIMA model exhibited superior predictive performance and was therefore selected as the final specification. The forecast horizon was restricted to December 2027, corresponding to 24 months beyond the end of the observed series. This two-year horizon was selected to provide a clinically and operationally relevant short-to-medium-term projection while limiting the accumulation of uncertainty associated with increasingly distant forecasts. Given the length of the available time series and the absence of external environmental predictors, forecasts substantially beyond two years were considered less reliable and were therefore not pursued. The 2026–2027 forecasts should consequently be interpreted as projections of the continuation of the observed seasonal dynamics rather than as long-term epidemiological predictions.

The SARIMA(1,0,1)(1,0,0)_12_ model was re-estimated using the full dataset (January 2016–December 2025) and used to generate out-of-sample forecasts for January 2026–December 2027.

Approximate 95% prediction intervals were computed as:

$${\hat{y}}_{t}\pm 1.96\cdot SE({\hat{y}}_{t}),$$

where $SE({\hat{y}}_{t})$ denotes the standard error of the forecast derived from the final model. Negative lower bounds were truncated at zero, reflecting the non-negative nature of incidence counts.

All analyses were performed using Stata 14 (StataCorp LLC, College Station, TX, USA) [11].

### 2.7. Model Diagnostic Tests

To assess model adequacy and verify underlying assumptions, a set of diagnostic tests was performed for both the SARIMA and the regression-based count models (Poisson and Negative Binomial).

#### 2.7.1. Residual Autocorrelation (Ljung–Box Test)

For the SARIMA model, residual autocorrelation was assessed using the Ljung–Box portmanteau test. This test evaluates whether residuals are independently distributed up to a specified lag order.

The null hypothesis is:

$$H_{0}:no\;autocorrelation\;up\;to\;lag\;h$$

The test statistic is:

$$Q=n(n+2)\sum_{k=1}^{h}\frac{{\hat{\rho }}_{k}^{2}}{n-k}$$

where ${\hat{\rho }}_{k}$ is the sample autocorrelation at lag k.

A non-significant result indicates that residuals behave as white noise, supporting model adequacy.

#### 2.7.2. Autocorrelation and Partial Autocorrelation Functions (ACF/PACF)

Residual diagnostics for the SARIMA model were further evaluated using the autocorrelation function (ACF) and the partial autocorrelation function (PACF). These plots were inspected to detect remaining temporal dependence.

Absence of significant spikes outside the confidence bounds suggests that the model adequately captures serial dependence in the data.

#### 2.7.3. Normality of Residuals (Skewness–Kurtosis Test)

The normality assumption of SARIMA residuals was assessed using the Skewness–Kurtosis test.

The null hypothesis is:

$$H_{0}:residuals\;are\;normally\;distributed$$

The test jointly evaluates deviations in skewness and kurtosis.

Although deviations from normality do not invalidate forecasting performance, they may indicate heavy tails or unmodelled structural variability.

#### 2.7.4. Goodness-of-Fit for Count Models

For Poisson and Negative Binomial models, overall goodness-of-fit was evaluated using Pearson chi-square statistics:

$$\chi^{2}=\sum \frac{{\left(y_{t}-{\hat{\mu }}_{t}\right)}^{2}}{{\hat{\mu }}_{t}}$$

A significant result suggests model misspecification or overdispersion.

In the presence of overdispersion, the Negative Binomial model relaxes the equidispersion assumption of the Poisson model by introducing an additional parameter α, such that:

$$Var(Y_{t})=\mu_{t}+\alpha \mu_{t}^{2}$$

#### 2.7.5. Overdispersion Test

The Poisson model assumes equality of mean and variance. This assumption was evaluated by comparing Poisson and Negative Binomial specifications.

A likelihood ratio test of:

$$H_{0}:\alpha =0$$

was performed. Rejection of the null supports the presence of overdispersion and justifies the Negative Binomial model.

#### 2.7.6. Residual Diagnostics for Count Models

For Poisson and Negative Binomial models, Pearson residuals were computed as:

$$r_{t}=\frac{y_{t}-{\hat{\mu }}_{t}}{\sqrt{Var(Y_{t})}}$$

These residuals were inspected using autocorrelation functions to assess any remaining serial dependence. Significant autocorrelation would indicate model misspecification in the temporal structure.

## 3. Results

**Figure 1:**
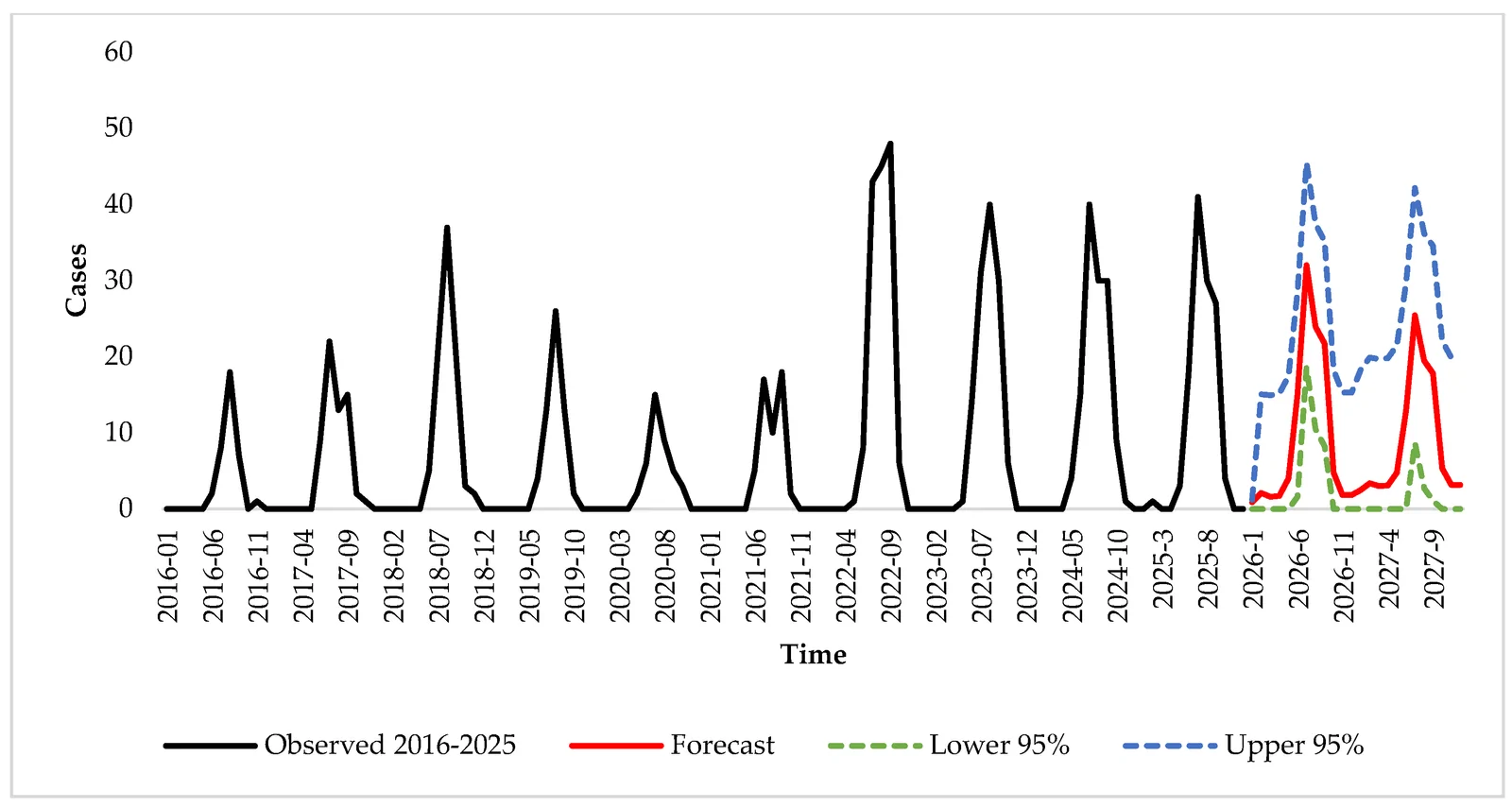
Observed monthly Toscana virus infections in Italy from January 2016 to December 2025 and SARIMA fitted values and forecasts for January 2026–December 2027, with 95% prediction intervals.

Three alternative modelling frameworks were evaluated: a SARIMA model, a Poisson regression model with seasonal harmonic terms, and a Negative Binomial regression model accounting for overdispersion. All models (see Table 2) were fitted to the training period from January 2016 to December 2023. The SARIMA(1,0,1)(1,0,0)_12_ model showed significant non-seasonal and seasonal autoregressive components. The AR(1) coefficient was positive and statistically significant (β = 0.489, 95% CI: 0.278–0.700, *p* < 0.001), as was the seasonal AR(1) component (β = 0.639, 95% CI: 0.493–0.786, *p* < 0.001). In contrast, the MA(1) coefficient was not statistically significant (β = 0.157, 95% CI: −0.118–0.466, *p* = 0.262). The estimated residual standard deviation was 6.062 (95% CI: 5.503–6.621, *p* < 0.001). Diagnostic assessment indicated that the SARIMA model adequately captured the temporal dependence in the series. The Ljung–Box test at lag 12 did not indicate significant residual autocorrelation (Q = 11.32, *p* = 0.350), while the residual ACF/PACF showed no evidence of remaining temporal structure. However, the skewness–kurtosis test rejected residual normality (*p* < 0.001), indicating that the residuals deviated from a Gaussian distribution. The Poisson regression model identified significant seasonal and lagged effects. Both harmonic terms were statistically significant, with coefficients of −2.592 (95% CI: −2.963 to −2.221, *p* < 0.001) for the sine component and −2.201 (95% CI: −2.464 to −1.938, *p* < 0.001) for the cosine component. The lagged outcome variable was also positively associated with the response (β = 0.022, 95% CI: 0.013–0.031, *p* < 0.001). However, the model exhibited overdispersion, as the observed variance exceeded the mean, and residual diagnostics indicated mild remaining temporal structure at lag 24. The Negative Binomial model similarly showed strong seasonal effects, with significant sine (β = −2.934, 95% CI: −3.487 to −2.381, *p* < 0.001) and cosine (β = −2.380, 95% CI: −2.759 to −2.001, *p* < 0.001) components. The lagged outcome remained statistically significant (β = 0.020, 95% CI: 0.004–0.035, *p* = 0.011). Importantly, the estimated overdispersion parameter was statistically significant (α = 0.143, 95% CI: 0.092–0.294, *p* < 0.001), confirming the presence of overdispersion and supporting the use of the Negative Binomial specification over the Poisson model. Residual diagnostics for the Negative Binomial model showed no relevant remaining autocorrelation at lag 24. Overall, these findings suggest that the SARIMA model provided an adequate representation of the temporal dependence, while the Negative Binomial model offered a more appropriate framework than Poisson regression for accounting for the overdispersed count data. Predictive accuracy was assessed using a hold-out sample covering January 2024–December 2025. The SARIMA model achieved the lowest mean absolute error (MAE = 4.75) and root mean squared error (RMSE = 7.63), compared with the Poisson model (MAE = 4.99, RMSE = 11.59) and the Negative Binomial model (MAE = 5.39, RMSE = 13.57) (Table 3). These results indicate a consistent advantage of the SARIMA specification in out-of-sample forecasting accuracy. Given its superior predictive performance, the SARIMA model was selected as the final forecasting approach and re-estimated using the full dataset (2016–2025), confirming a stable and highly persistent seasonal structure consistent with the observed annual cycles. Observed incidence alongside SARIMA-based fitted values and forecasts for 2026–2027 are shown in Figure 1 and Table 4. The model closely reproduces the main seasonal oscillations observed during the historical period and projects their continuation over the forecast horizon. The 95% prediction intervals widen progressively with increasing forecast horizon, reflecting the accumulation of predictive uncertainty. The forecasts should therefore be interpreted as an expected continuation of the historical seasonal pattern rather than as a deterministic prediction of future incidence.

## 4. Discussion

### 4.1. Epidemiological and Seasonal Dynamics of Toscana Virus

The present study confirms that TOSV in Italy follows a highly reproducible seasonal pattern, with consistent summer peaks over a decade-long observation period. This aligns with established evidence that TOSV is a sandfly-borne phlebovirus whose transmission is tightly linked to the seasonal dynamics of Phlebotomus vectors [3,9]. TOSV is an important cause of arthropod-borne central nervous system infections in endemic Mediterranean regions, particularly during summer months when vector abundance is highest [9,12]. The stable seasonality observed in this study suggests that the ecological niche of TOSV in Italy has remained unchanged over time, consistent with long-term surveillance studies in central Italy [3,9,12]. Seroprevalence studies further indicate that infection is widespread in endemic regions, often underdiagnosed due to asymptomatic or mild clinical presentation [4], reinforcing the concept that reported cases likely represent only a fraction of true infections.

### 4.2. Vector Ecology and Environmental Drivers

The seasonal dynamics of TOSV are primarily driven by the biology of sandfly vectors, whose abundance is strongly influenced by temperature, humidity, and habitat suitability [5,12,13]. Studies on Phlebotomus perniciosus and related species demonstrate that transmission peaks coincide with optimal climatic conditions for vector survival and viral replication [5,12,13,14]. In Italy and other Mediterranean countries, climate variability has been associated with changes in sandfly distribution and density, which in turn affects arbovirus transmission intensity [13,15]. These ecological constraints explain the strong annual periodicity observed in TOSV incidence. More recent epidemiological syntheses confirm that sandfly-borne phleboviruses are expanding their geographic range in Europe, potentially influenced by environmental and anthropogenic changes [13,15].

### 4.3. Comparison of SARIMA- and Regression-Based Models

The superior performance of SARIMA compared with the Poisson and Negative Binomial models in our validation analysis reflects the strong temporal dependence and seasonality observed in the TOSV series. This finding is also conceptually consistent with previous infectious disease forecasting studies showing that models explicitly incorporating temporal and seasonal structure can provide useful short-term forecasts when disease incidence exhibits recurrent seasonal patterns. In contrast, studies such as those by Nicoletti et al. [13] and Wang et al. [15] primarily provide epidemiological and ecological evidence concerning sandfly-borne viruses and the environmental determinants of phlebotomine sandfly distribution, rather than direct evidence comparing forecasting algorithms. In our analysis, the Poisson and Negative Binomial models incorporated harmonic seasonal terms and lagged incidence, but these specifications did not fully capture the temporal dependence present in the monthly TOSV series. The superior predictive performance of SARIMA therefore suggests that explicitly modelling serial correlation and seasonal autoregressive structure may be particularly useful for short-term forecasting of TOSV incidence. The finding should nevertheless be interpreted in the context of the present validation design and should not be generalized to all infectious disease forecasting settings. Even when harmonic seasonal terms and lagged incidence are included, count-based regression models may fail to fully represent complex stochastic dependencies [16,17]. This finding is consistent with previous applications of statistical modelling to infectious disease surveillance data, where time series models can provide useful short-term predictions when strong temporal and seasonal patterns are present [13,15].

### 4.4. Overdispersion and Stochastic Structure of Incidence Data

The significant overdispersion observed in the Negative Binomial model confirms heterogeneity in TOSV incidence. Overdispersion in infectious disease data is commonly attributed to unobserved variability in exposure risk, clustering of cases, and environmental heterogeneity [17]. However, correcting for overdispersion alone did not improve predictive accuracy compared to SARIMA, suggesting that temporal dependence rather than distributional variance is the primary driver of model performance differences. This pattern has been observed in other vector-borne diseases, where Negative Binomial models improve fit but do not necessarily enhance forecasting ability [15,18].

### 4.5. Public Health Implications

Predictive approaches may also contribute to risk stratification when combined with epidemiological, environmental, meteorological, entomological, and geographical information. In particular, periods characterized by increased predicted incidence could be used to support intensified epidemiological and entomological surveillance, targeted monitoring of sandfly populations, and increased clinical awareness in areas considered at higher risk. At the health-system level, forecasts could support prospective allocation of laboratory capacity, diagnostic resources, and clinical preparedness during periods in which an increase in neuroinvasive infections is anticipated. Such information may be particularly useful in endemic areas with limited resources, where anticipatory allocation may be more efficient than responding only after a substantial increase in reported cases. Comparable predictive and early-warning approaches have been developed for other vector-borne infections. The World Health Organization has supported the development and implementation of the Early Warning and Response System (EWARS) for climate-sensitive diseases, a data-driven predictive framework that uses historical health data and environmental indicators to identify signals of forthcoming outbreaks and support timely response. The EWARS framework has been applied to dengue and other climate-sensitive diseases, illustrating how predictive information can be incorporated into surveillance systems to support earlier outbreak detection and public health preparedness [19,20]. For West Nile virus (WNV), predictive modelling has similarly been investigated as a component of early-warning strategies, using environmental and climatic information to anticipate periods of increased transmission risk and to inform preparedness and vector-control planning [21,22]. Particularly relevant to the Italian context, a recent study in Northern Italy developed a spatial-temporal Bayesian model for West Nile neuroinvasive disease and found that temperature recorded one to two months before case occurrence was strongly associated with subsequent incidence. The temperature-based model achieved an AUC of 0.81 when evaluated using out-of-sample data and was proposed as a potential early-warning tool for anticipating periods of increased WNV neuroinvasive disease risk [22]. These examples illustrate the potential translational value of predictive modelling for vector-borne diseases. Rather than replacing established epidemiological and entomological surveillance, forecasting can provide an anticipatory layer that transforms routinely collected information into actionable risk signals. In the context of TOSV, a forecast of an approaching seasonal increase could therefore support earlier clinical awareness, targeted vector surveillance, laboratory preparedness, and more timely allocation of healthcare resources. In endemic regions, this approach could help optimize preparedness around predictable seasonal peaks, whereas in emerging or newly affected areas it could potentially be adapted by incorporating spatial, climatic, and entomological information to identify areas where transmission risk is increasing. However, the present study evaluates predictive accuracy rather than the effectiveness of forecast-driven interventions. Therefore, the potential benefits described above should be considered translational opportunities rather than demonstrated effects on outbreak control or patient outcomes. Prospective studies are needed to determine whether integrating TOSV forecasts into routine surveillance can improve the timeliness of detection, optimize resource allocation, and ultimately contribute to improved public health outcomes.

### 4.6. Methodological Considerations

From a methodological perspective, SARIMA models remain highly effective for short-term forecasting in systems with stable seasonal dynamics. Their strength lies in capturing autocorrelation structures without requiring explicit covariates [16]. However, more flexible modelling approaches, including mechanistic and hybrid statistical models, are increasingly being explored for infectious disease forecasting [13,18]. These approaches may offer advantages in systems where structural changes or nonlinear effects are more prominent. In the case of TOSV, the stability of temporal dynamics suggests that relatively simple stochastic models remain appropriate for operational forecasting.

### 4.7. Limitations

This study has several limitations. Surveillance data may underestimate true incidence due to underdiagnosis and asymptomatic infections, a known issue in TOSV epidemiology [4]. In addition, the absence of environmental covariates limits mechanistic interpretation of transmission dynamics. The use of monthly aggregation may obscure short-term fluctuations in transmission, while the single split validation approach may not fully capture temporal variability in model performance. Finally, SARIMA models assume a linear structure and may not capture potential nonlinear effects or abrupt shifts in transmission dynamics driven by environmental or ecological changes. Moreover, not all possible model specifications were explored.

## 5. Conclusions

This study demonstrates that Toscana virus (TOSV) incidence in Italy exhibits a stable and pronounced seasonal pattern over the 2016–2025 period. Among the evaluated models, the SARIMA(1,0,1)(1,0,0)_12_ specification showed the best out-of-sample predictive performance, outperforming both Poisson and Negative Binomial regression models during the 2024–2025 validation period.

These findings indicate that temporal dependence and seasonal structure are important components of short-term TOSV forecasting. SARIMA-based forecasts may therefore complement routine surveillance by providing anticipatory information on expected seasonal increases in reported incidence, although their operational value for public health decision-making requires prospective evaluation.

Future studies should investigate whether incorporating meteorological, environmental, entomological, and spatial information can improve predictive performance and provide more geographically resolved risk estimates. Comparative evaluation of SARIMA, hybrid, and machine learning approaches using rolling or prospective validation would also help determine the most robust strategies for developing operational early-warning systems for TOSV and other emerging vector-borne infections in Mediterranean regions.

## Figures and Tables

**Table 1:** Comparison of SARIMA Models and Their Performance Metrics.

SARIMA Models	AIC	BIC	MAE	RMSE
(1,0,0)(1,0,0)_12_	630.52	635.67	4.81	7.74
(1,0,1)(1,0,0)_12_	631.20	638.92	4.75	7.63
(1,0,0)(1,0,1)_12_	632.52	640.24	4.87	7.79
(1,0,1)(1,0,1)_12_	632.94	643.23	4.81	7.69

**Table 2:** Model Estimates and Diagnostic Evaluation.

Model	Variable/Test	Estimate (95% CI)	*p*-Value	Diagnostics
SARIMA(1,0,1)(1,0,0)_12_	AR(1)	0.489 (0.278–0.700)	<0.001	
	MA(1)	0.157 (−0.118–0.466)	0.262	
	Seasonal AR(1)	0.639 (0.493–0.786)	<0.001	
	Sigma	6.062 (5.503–6.621)	<0.001	
	Ljung–Box Q (lag 12)	11.32	0.350	No residual autocorrelation
	Skewness–Kurtosis test	37.84	<0.001	Non-normal residuals
	ACF/PACF residuals	–	–	No remaining structure
	t	0.007 (0.004−0.011)	<0.001	
Poisson regression	sin(2πt/12)	−2.592 (−2.963–−2.221)	<0.001	
	cos(2πt/12)	−2.201 (−2.464–−1.938)	<0.001	
	Lag Y(t − 1)	0.022 (0.013–0.031)	<0.001	
	Intercept	−0.852 (−1.221–−0.484)	<0.001	
	Overdispersion	–	–	Present (variance > mean)
	Residual ACF (lag 24)	–	–	Mild residual structure
	t	0.008 (0.002−0.014)	0.003	
Negative Binomial	sin(2πt/12)	−2.934 (−3.487–−2.381)	<0.001	
	cos(2πt/12)	−2.380 (−2.759–−2.001)	<0.001	
	Lag Y(t − 1)	0.020 (0.004–0.035)	0.011	
	Intercept	−1.162 (−1.693–−0.631)	<0.001	
	Overdispersion (α)	0.143 (0.092–0.294)	<0.001	Overdispersion significant
	Pearson residual ACF (lag 24)	–	–	No relevant structure

**Table 3:** Forecast accuracy in validation period (2024–2025).

Model	MAE	RMSE
SARIMA	4.75	7.63
Poisson regression	4.99	11.59
Negative Binomial regression	5.39	13.57

**Table 4:** Monthly SARIMA forecasts of Toscana virus infections in Italy, 2026–2027.

Month	Forecast	95% Prediction Interval
January 2026	0.88	0.00–12.15
February 2026	2.10	0.00–15.07
March 2026	1.59	0.00–14.95
April 2026	1.71	0.00–15.15
May 2026	3.98	0.00–17.44
June 2026	15.06	1.59–28.53
July 2026	32.03	18.56–45.50
August 2026	23.93	10.46–37.40
September 2026	21.72	8.25–35.19
October 2026	4.77	0.00–18.24
November 2026	1.82	0.00–15.29
December 2026	1.82	0.00–15.29
January 2027	2.47	0.00–18.30
February 2027	3.36	0.00–19.89
March 2027	2.99	0.00–19.68
April 2027	3.08	0.00–19.80
May 2027	4.75	0.00–21.49
June 2027	12.92	0.00–29.66
July 2027	25.43	8.70–37.17
August 2027	19.46	2.72–36.20
September 2027	17.83	1.09–34.57
October 2027	5.33	0.00–22.07
November 2027	3.16	0.00–19.90
December 2027	3.16	0.00–19.90

## Data Availability

The data are public and available on the website of the Italian National Institute of Health.
